# Biocontrol Potential of Entomopathogenic Fungi Against Plant-Parasitic Nematodes: A *Caenorhabditis elegans*-Based Screening and Mechanistic Study

**DOI:** 10.3390/jof11050381

**Published:** 2025-05-16

**Authors:** Cheng Cheng, Renjun Zhang, Yanzhen Wang, Shuo Yang, Wenhao Yu, Yuxian Xia

**Affiliations:** 1 Genetic Engineering Research Center, School of Life Sciences, Chongqing University, Chongqing 401331, China; 2 Chongqing Engineering Research Center for Fungal Insecticide, Chongqing 401331, China; 3 Key Laboratory of Gene Function and Regulation Technologies under Chongqing Municipal Education Commission, Chongqing 401331, China; 4 School of Pharmacy, Chongqing University, Chongqing 401331, China

**Keywords:** nematode, *Metarhizium anisopliae*, *Meloidogyne incognita*, *Caenorhabditis elegans*, nematocidal activity, biopesticides

## Abstract

Plant-parasitic nematodes and insect pests critically threaten agricultural productivity, but chemical pesticides face limitations due to resistance and environmental concerns, necessitating eco-friendly biopesticides targeting both pests and nematodes. Here, we developed a high-throughput screening platform using *Caenorhabditis elegans* to identify entomopathogenic fungi exhibiting nematocidal activity against *Meloidogyne incognita*. Among 32 tested strains, nine *Metarhizium* spp. and one *Beauveria* strain demonstrated dual efficacy against *C. elegans* and *M. incognita*. *Metarhizium anisopliae* CQMa421 showed the highest virulence, suppressing nematode reproduction by 42.7% and inducing >80% mortality. Pot experiments revealed a 50% reduction in the root galling index and 50.3% fewer root galls in *Solanum lycopersicum*. The CQMa421 filtrate caused irreversible locomotor deficits and reduced egg hatching rates by 28%. Concurrently, intestinal damage, elevated oxidative stress and autophagy were observed in *C. elegans*. This was accompanied by a transcriptome-wide modulation of genes involved in detoxification and immune defense pathways. These findings demonstrate the efficacy of our *C. elegans*-based screening method for identifying fungi with nematocidal potential. CQMa421’s virulence against *M. incognita* suggests its promise for pest management, while molecular insights highlight pathways that may contribute to the future design of future nematicides. This study advances fungal biocontrol agents and offers a sustainable strategy for agriculture.

## 1. Introduction

Insect pests and plant-parasitic nematodes pose significant threats to global agricultural productivity, reducing crop yields, compromising plant health, and exacerbating economic losses that undermine food security [1]. Over 4100 species of plant-parasitic nematodes have been documented, with *Meloidogyne incognita* (Kofoid and White, 1912) Chitwood standing out as one of the most destructive due to its broad host range, rapid reproduction and adaptability [2,3]. The current reliance on chemical nematicides for control has led to environmental contamination, human health risks and the emergence of resistant pest populations [4,5]. These challenges highlight the urgent need for sustainable alternatives, such as microbial biocontrol agents. While bacteria like *Enterobacter asburiae* Brenner et al., 1988 and fungi such as *Trichoderma* spp., *Arthrobotrys oligospora* Fresenius 1850, and *Purpureocillium lilacinum* (Thom) Luangsa-ard, Houbraken, Hywel-Jones & Samson (2011) exhibit nematocidal activity, they lack the capacity to simultaneously target insect pests [6,7,8].

Entomopathogenic fungi, such as *Metarhizium* and *Beauveria* spp., offer a sustainable solution for pest management. These fungi are widely employed in biocontrol due to their safety for non-target organisms, plant colonization capacity and the production of bioactive secondary metabolites [9,10,11,12]. Their dual role in suppressing pests and enhancing plant growth further positions them as ideal candidates for integrated pest management [13]. While prior studies highlight the biocontrol potential of specific *Metarhizium* and *Beauveria* strains [14,15], a critical gap remains. The lack of rapid and practical methods to screen strains for nematocidal activity limits their widespread use in sustainable agriculture.

Traditional methods for screening nematocidal fungi rely on labor-intensive plant-based assays or nematode cultivation, which are limited by long growth cycles and the complex life stages of plant-parasitic nematodes like *M. incognita* [3,16]. In contrast, the model nematode *Caenorhabditis elegans* (Maupas, 1900) Dougherty 1955 provides a tractable system for high-throughput screening due to its short life cycle, genetic transparency and well-characterized molecular pathways [17,18]. Furthermore, *C. elegans* serves as a surrogate for studying plant-parasitic nematode biology, enabling mechanistic insights into fungal–nematode interactions [19,20]. However, key physiological and ecological distinctions between *C. elegans* and *M. incognita* must be acknowledged. Unlike *M. incognita*, which exhibits complex host interaction cycles involving specialized feeding structures, *C. elegans* lacks the anatomical adaptations for plant tissue penetration and intracellular parasitism. Additionally, *M. incognita* exhibits sexual dimorphism and soil-dwelling infective juvenile stages—features absent in the self-fertilizing hermaphroditic model nematode [21]. Despite these differences, conserved molecular pathways governing detoxification (e.g., nuclear hormone receptors), neurotoxicity (e.g., acetylcholinesterases) and immune responses are shared across nematode clades [20,22,23]. This evolutionary conservation supports the use of *C. elegans* for the preliminary screening of nematocidal agents.

Here, we evaluated 32 *Metarhizium* and *Beauveria* strains for nematocidal activity using *C. elegans*. Among these, the CQMa421 filtrate emerged as the most potent against both *C. elegans* and *M. incognita*. The CQMa421 culture filtrate disrupted nematode locomotion, reproduction and intestinal integrity, while a transcriptomic analysis revealed the modulation of detoxification, immune defense and oxidative stress pathways. This study establishes *C. elegans* as a tool for the rapid screening of entomopathogenic fungi and advances CQMa421 as a potential candidate for pest and nematode management.

## 2. Materials and Methods

### 2.1. Materials and Maintenance

Thirty-two entomopathogenic fungal strains (16 *Metarhizium* spp. strains: GB31.000127, GB31.000128, GB3100289, GB3100290, GB3100293, GB3100311–GB3100315 and GB3100317–GB3100322; 16 *Beauveria* spp. strains: GB3200001–GB3200011, GB3200023 and GB3200028–GB3200031) were obtained from the Genetic Engineering Research Center, Chongqing University. Bristol N2 and MAH215[*lgg-1p::mcherry::gfp::lgg-1*] *Caenorhabditis elegans* strains were provided by Dr. Pang Shan-Shan from Chongqing University. *Meloidogyne incognita* second-stage juveniles (J2s) were isolated from infected tomato (*Solanum lycopersicum* L.) roots grown in a greenhouse in Chongqing, China. Tomato seedlings were obtained from the Chinese Academy of Agricultural Sciences (Bishan, Chongqing, China) and cultivated in nutrient soil (Pindstrup Mosebrug A/S, Ryomgaard, Denmark).

### 2.2. Preparation of Culture Filtrates and Nematodes

Fungal strains were cultured in 1/4 soy dextrose yeast (SDY) media (10 g/L dextrose, 2.5 g/L peptone and 5 g/L yeast extract; pH 6.4) inoculated with 5 × 10^6^ conidia/mL and incubated at 220 rpm and 28 °C for 48 h. Culture filtrates were obtained by centrifugation at 12,000 rpm for 20 min at 4 °C, and then sterile filtration through a 0.22 μm membrane. *M. incognita* J2s were extracted from cysts dissected from tomato roots. *C. elegans* was synchronized using a 5% bleach solution, and the first-stage larvae (L1) were cultured on nematode growth medium (NGM) plates seeded with *Escherichia coli* (Migula 1895) Castellani and Chalmers 1919, strain: OP-50 at 20 °C to obtain L4-stage larvae (L4), young adults (YA) and day-1 adults (D1). The NGM culture medium was prepared with 3 g/L NaCl, 2.5 g/L peptone, 17 g/L agar, 1 mM CaCl_2_, 1 mM MgSO_4_, 25 mM potassium phosphate buffer (at a pH of 6.0) and 5 μg/mL cholesterol.

### 2.3. Pot Experiment

Three-week-old tomato seedlings were transplanted into pots (with a 15.5 cm diameter) containing a 1:1 mixture of nutrient soil and *M. incognita*-infested soil (>500 J2s per 20 g). Plants were inoculated twice weekly with 20 mL of CQMa421 filtrate at four points (1 cm from the root system). The experiment included 10 replicates per treatment and was repeated 3 times under controlled greenhouse conditions (at 25 °C and 65% relative humidity) with regular watering. Stem parameters (length, diameter and fresh/dry weight) were assessed 21 days after inoculation. After 60 days, root parameters (length and fresh/dry weight) and galling severity were assessed using the Hussey and Janssen scale [24], with gall counts normalized per 5 g of roots for standardized analysis.

### 2.4. Reproduction and Lethality Assays

Nematode mortality and hatching assays were adapted from established protocols [25]. Briefly, 50 μL of fungal filtrate (or 1/4 SDY control) was added to 96-well plates containing approximately 50 *C. elegans* or *M. incognita* J2s. Mortality and hatching rates were recorded after 24 h at 20 °C. Pearson’s correlation analysis assessed the nematocidal activity of 32 strains on *M. incognita* and *C. elegans* using R software (version 4.5.0). For egg-laying assays [26], 10 D1 adults were exposed to 50 μL CQMa421 filtrate for 6 h at 20 °C, and hourly egg production was quantified. All assays included ≥6 replicates per treatment and were repeated three times. Nematode viability was assessed via a Mitoc^TM^ SMZ-168 Stereozoom^TM^ microscope (Kowloon Bay, Hong Kong), with individuals exhibiting rigidity and unresponsiveness to stimulation classified as dead.

### 2.5. Locomotor Behaviour Assays

D1 adults treated with CQMa421 filtrate were transferred to NGM plates seeded with *E. coli* OP50 for pharyngeal pumping analysis or to unseeded plates for head thrash quantification [27]. Each terminal bulb movement cycle was recorded as a single pumping event. A head thrash was defined as a body bend exceeding half the nematode’s length. For the recovery assays, treated worms were incubated on *E. coli* OP50-seeded plates for 12 h before measurement. Ten nematodes per group were analyzed in triplicate.

### 2.6. Oxidative Stress, Intestinal Damage and Autophagy Evaluation

Intracellular ROS in *C. elegans* was measured using 10 μM H_2_DCFDA at 20 °C for 2 h [28]. Intestinal lipid droplets were stained with Nile red (1 μg/mL) at 20 °C for 2 h to assess damage [29]. To avoid egg interference, L4 worms were used in ROS and intestinal evaluation. Following incubation, the worms were washed, anaesthetized with 2 mM levamisole hydrochloride and mounted on 2% agarose slides for visualization under a fluorescence microscope (Nikon^®^ Eclipse 80i, Tokyo, Japan). For autophagy [30], MAH215[*lgg-1p::mcherry::gfp::lgg-1*] D1 adults were exposed to CQMa421 filtrate for 6 h, and autophagosomes (GFP^+^/mCherry^+^) and autolysosomes (GFP^−^/mCherry^+^) were quantified via confocal microscopy (Leica^®^ TCS SP8 CSU, Wetzlar, Germany). For each group, 30 nematodes were analyzed and quantified via ImageJ software (version 1.53t, U.S. National Institutes of Health).

### 2.7. Transcriptome and qRT-PCR Analysis

Transcriptomic analysis was conducted for D1-stage *C. elegans* treated with CQMa421 culture filtrate (treatment) or 1/4 SDY medium (control) for 6 h, a time point selected based on the observed phenotypic and metabolic alterations. RNA sequencing was performed by Beijing Genomics Institute (BGI), utilizing the *C. elegans* reference genome (NCBI/GCF_000002985.6_WBcel235). Raw data have been deposited in the NCBI Sequence Read Archive (accession number: PRJNA1160025). Differentially expressed genes (DEGs) were identified using DESeq2 with thresholds of |log_2_(fold change)| ≥ 1 and Q value ≤ 0.05. DEGs were classified into gene families based on functional annotations and subsequently categorized into biological roles using Gene Ontology (GO) and Kyoto Encyclopedia of Genes and Genomes (KEGG) analyses. Protein–protein interaction (PPI) networks were constructed using the STRING database and NCBI reference transcripts to analyze associations among DEGs. To correlate filtrate effects with key genes, DEGs were systematically linked to filtrate-induced phenotypic and metabolic alterations.

Transcriptome results were validated using qRT-PCR (Figure S1), following the protocol of Tang and Pang [31]. Each biological replicate was assayed in duplicate, with data normalized to the *snb-1* gene. Primer sequences for *C. elegans* genes are listed in Supplementary Table S1.

### 2.8. Data Analysis

Data were analyzed using IBM^®^ SPSS^®^ 23.0 and visualized in GraphPad^®^ Prism^®^ 7. Images were processed with Adobe^®^ Photoshop^®^ CS6 software. For nematocidal activity screening, lethality metrics (mean, SD and median) were computed per strain (Table 1), followed by non-parametric Kruskal–Wallis testing (*p* < 0.001) with five defined tiers (letter group a–e; overlapping letters indicate it is non-significant via Dunn’s test at *p* > 0.05). Normality (Shapiro–Wilk; α = 0.05) and variance homogeneity (Levene’s test; α = 0.05) guided statistical selection: Pearson/Spearman correlations were used for the bivariate analysis (scatterplots annotated with coefficients/*p* values) and a tiered workflow was used for other data—non-normal distributions (*p* < 0.05) used Mann–Whitney U test, normally distributed but heteroscedastic data employed Welch’s *t*-test, and normal homoscedastic data utilized Student’s *t*-test. All outputs are cataloged in Table S2.

## 3. Results

### 3.1. Evaluation of a C. elegans-Based Screening Platform

To evaluate the feasibility of using *C. elegans* as a screening platform for identifying entomopathogenic fungi with nematocidal activity against *M. incognita*, we tested 16 *Metarhizium* spp. and 16 *Beauveria* spp. strains against both nematodes. Non-normal distributions in both groups (via the Shapiro–Wilk test at *p* < 0.05 for both *C. elegans* and *M. incognita*) necessitated Spearman’s rank correlation analysis. The analysis of 32 strains demonstrated a significant correlation between lethal rates on both nematode species (*ρ* = 0.5236, 95% confidence interval [0.2037, 0.7425], *p* = 0.0021). Nine *Metarhizium* strains (GB31.000128, GB3100289, GB3100290, GB3100313, GB3100314, GB3100315, GB3100318, GB3100319 and GB3100322) exhibited high virulence against *C. elegans*, with mortality rates ranging from 44.44% to 85.71% (Table 1). However, *Beauveria* strains showed limited activity, with the most effective strain (GB3200028) inducing only 9.09% mortality. Notably, seven of nine *C. elegans*-lethal *Metarhizium* strains also demonstrated significant activity against *M. incognita*, and 20 of 22 strains non-lethal to *C. elegans* were similarly ineffective against *M. incognita* (Table 1). These findings validate *C. elegans* as a rapid and reliable tool for identifying entomopathogenic fungi with biocontrol potential against nematodes.

**Figure 1:**
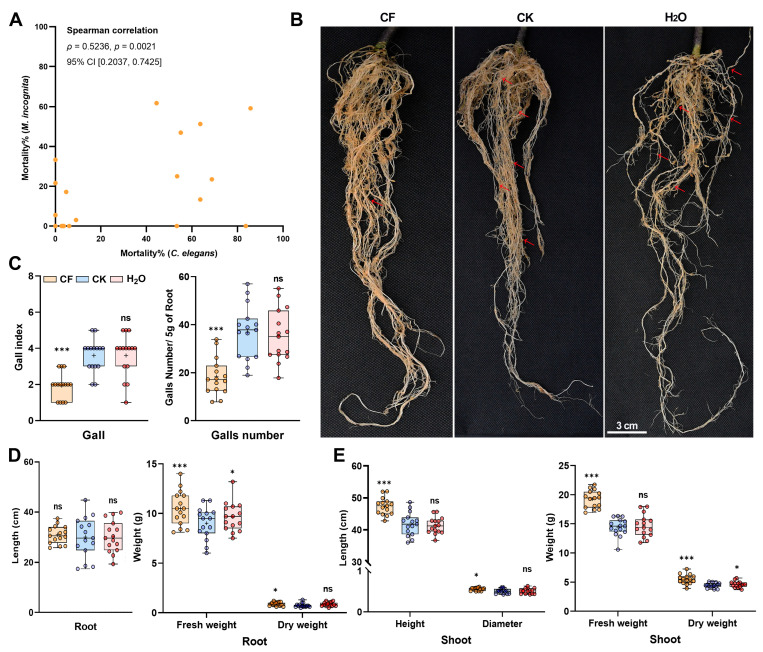
(**A**) Correlation between lethality of entomopathogenic fungal filtrates in *C. elegans* and *M. incognita*. (**B**) Representative root systems after treatment: CF (CQMa421 filtrate), CK (1/4 SDY control), H_2_O (blank control); red arrows denote *M. incognita* egg masses. (**C**) Root gall index and gall counts per 5 g of roots. (**D**) Root length and fresh/dry weights. (**E**) Stem height, diameter and fresh/dry weights. Root and stem parameters were assessed 60 and 21 days post-treatment, respectively. Boxplots show the range from minimum to maximum. The central line indicates the median, and the ‘+’ symbol represents the mean. Statistical test used can be found in Table S2. * *p* < 0.05, *** *p* < 0.001, ns indicates non-significant.

Among the tested strains, *Metarhizium anisopliae* CQMa421 (GB3100289, CGMCC No. 460) displayed the highest nematocidal activity against both nematodes, achieving 85.71% mortality in *C. elegans* and marked efficacy against *M. incognita*. Pot experiments demonstrated that the CQMa421 filtrate significantly alleviated *M. incognita*-induced damage in *Solanum lycopersicum* (Figure 1B–E). The root galling index decreased by 50%, from 4.0 to 2.0 (*p* < 0.0001), and the number of galls per 5 g of root declined by 50.3%, from 36.867 to 18.334 (*p* = 0.0004), demonstrating the effective suppression of nematode parasitism. Concurrently, root health improved substantially, with fresh weight increasing by 16.8% (*p* = 0.001) and dry weight increasing by 28.6% (*p* = 0.048), while root length showed no significant differences (*p* = 0.1728). Aboveground, plant height increased by 13.5%, from 41.774 cm to 47.4 cm (*p* < 0.0001), accompanied by a 14.1% increase in stem diameter (*p* = 0.0279), while shoot biomass demonstrated substantial gains, with fresh weight increasing by 33.3% and dry weight increasing by 24.4%. These findings underscore the dual role of the CQMa421 filtrate in mitigating nematode-induced damage and promoting plant vigor through enhanced biomass allocation, positioning it as a promising biocontrol agent for pest management. Thus, CQMa421 was prioritized to investigate its nematotoxic effects and elucidate the molecular mechanisms underlying its toxicity in *C. elegans*.

### 3.2. Effects of the CQMa421 Culture Filtrate on C. elegans Survival, Locomotion and Reproduction

**Figure 2:**
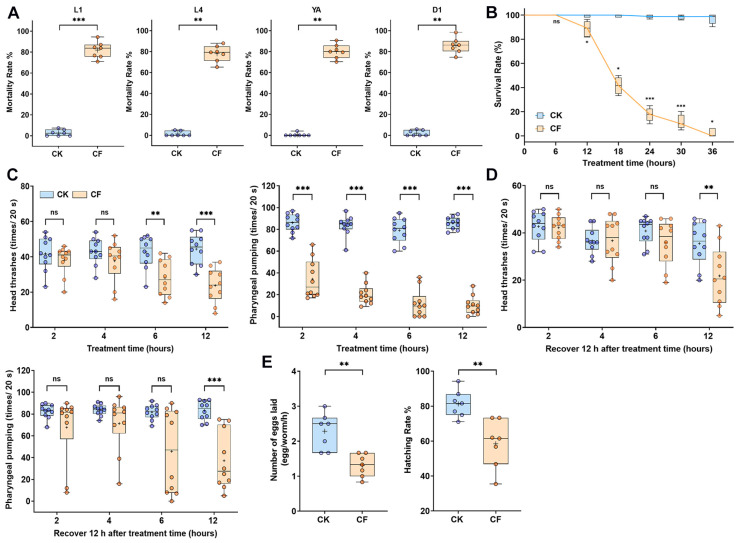
Effects of CQMa421 filtrate on *C. elegans* survival, locomotion, and reproduction. (**A**) Mortality rates of L1, L4, young adult (YA) and day 1 adult (D1) nematodes after 24 h exposure. (**B**) Survival curve of D1 adults. (**C**) Head thrashes and pharyngeal pumping frequency in D1 adults. (**D**) Recovery of locomotor behavior 12 h post-treatment. (**E**) Egg-laying rate and hatching efficiency. CK: 1/4 SDY control, CF: CQMa421 filtrate. Boxplots show the range from minimum to maximum. The central line indicates the median, and the ‘+’ symbol represents the mean. Statistical test used can be found in Table S2. * *p* < 0.05, ** *p* < 0.01, *** *p* < 0.001, ns indicates non-significant.

To evaluate the phenotypic effects of the CQMa421 culture filtrate on nematodes, mortality rates, locomotor behavior and reproductive metrics were assessed in *C. elegans*. The filtrate exhibited multi-stage nematocidal activity, with mortality rates of 81.93%, 79.31%, 80% and 86.28% for L1, L4, YA and D1 worms, respectively (Figure 2A). D1 adults, showing the highest mortality, were selected for subsequent assays. During observations, although no statistically significant difference in mortality was observed after 6 h of exposure, both pharyngeal pumping and head thrashes were severely disrupted by the CQMa421 filtrate (Figure 2B,C). Pharyngeal pumping decreased significantly within 2 h (*p* < 0.0001), declining to 9.5 pumps/20 s after 6 h, compared to 81 pumps/20 s in the control. Unlike pharyngeal changes, head thrashes remained unaffected until reaching 6 h of treatment. These locomotor behaviors continuously decreased with increasing exposure time (Figure 2C). To assess whether the observed decline could be reversed, treated nematodes were recovered by transferring them to NGM plates with or without *E. coli* OP50 for 12 h before counting. Recovery assays revealed that brief exposures of 2–6 h resulted in a partial restoration of locomotion, whereas prolonged treatment (12 h) induced irreversible damage to the nematodes (Figure 2D), demonstrating the cumulative and irreversible nature of damage caused by the CQMa421 culture filtrate.

To assess reproductive toxicity, both egg-laying numbers and egg hatching rates were quantified. CQMa421 filtrate exposure reduced the number of eggs laid by 42.7%, corresponding to an average reduction of one egg per hour (*p* = 0.0013). Furthermore, the hatching rate of the eggs decreased significantly (*p* = 0.008), exhibiting a 28.0% reduction compared to the control (Figure 2E). These results demonstrate that the CQMa421 filtrate exhibits multifaceted nematocidal activity, causing stage-specific mortality, irreversible locomotor deficits and reproductive toxicity in *C. elegans*.

### 3.3. CQMa421 Culture Filtrate Induces Intestinal Damage, Oxidative Stress and Autophagy in C. elegans

**Figure 3:**
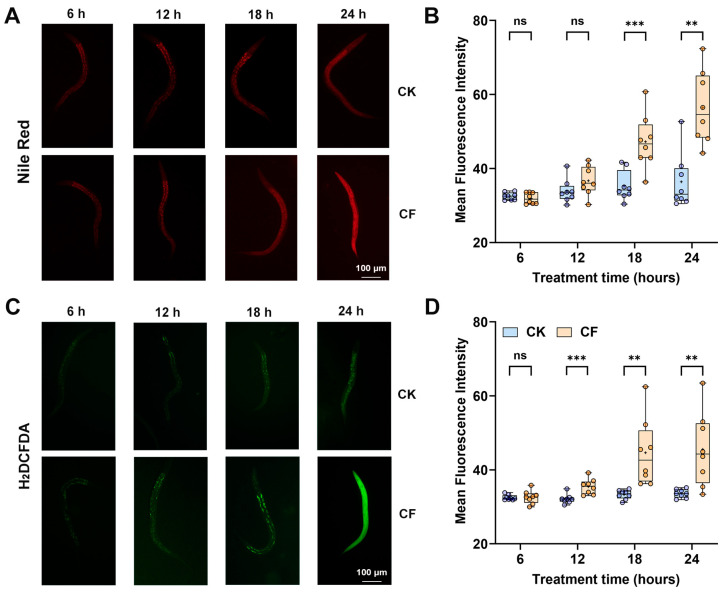
CQMa421-filtrate-induced intestinal damage and ROS accumulation in *C. elegans*. (**A**) Nile-red-stained intestinal lipid droplets. (**B**) Quantification of Nile red fluorescence. (**C**) H_2_DCFDA-stained ROS levels. (**D**) Quantification of ROS fluorescence. CK: 1/4 SDY control, CF: CQMa421 filtrate. Boxplots show the range from minimum to maximum. The central line indicates the median, and the ‘+’ symbol represents the mean. Statistical test used can be found in Table S2. ** *p* < 0.01, *** *p* < 0.001, ns indicates non-significant.

To investigate the tissue and metabolic changes in *C. elegans* caused by the CQMa421 filtrate, the intestinal barrier, reactive oxygen species (ROS) levels and autophagy were assessed using dye reagents and transgenic nematodes. After 18 h of filtrate exposure, significant intestinal damage was observed (*p* = 0.0002), marked by an extensive lipid accumulation area and staining diffusion from the intestine to the entire body (Figure 3A,B). Similar results were observed for ROS accumulation in nematodes, with a pronounced and statistically significant increase in ROS production (*p* = 0.0007) compared to the control after 12 h of treatment (Figure 3C,D). ROS accumulation was consistent with the intestinal staining area, mainly concentrated in the fat deposits surrounding the intestine.

**Figure 4:**
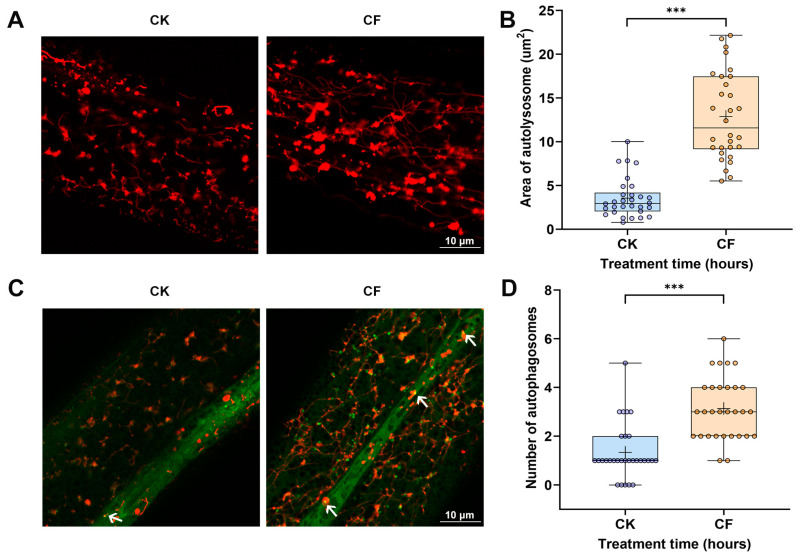
Modulation of autophagy by CQMa421 filtrate treatment. (**A**,**B**) Area of autolysosome and quantification. (**C**,**D**) Number of autophagosomes. Markers: red (mCherry for autolysosomes), green (GFP), yellow (GFP + mCherry overlap). Arrows represent autophagosomes. Scale bar = 10 μm. CK: 1/4 SDY control, CF: CQMa421 culture filtrate. Boxplots show the range from minimum to maximum. The central line indicates the median, and the ‘+’ symbol represents the mean. Mann–Whitney U, *** *p* < 0.001.

To evaluate autophagy, transgenic *C. elegans* expressing mCherry::GFP::lgg-1 fusion protein showed that filtrate exposure significantly increased autophagosome counts and autolysosome areas (*p* < 0.0001), indicating enhanced autophagic flux (Figure 4). In summary, filtrate treatment disrupted the normal physiological metabolism of nematodes, causing cell damage and organ dysfunction. Intestinal damage, increased ROS levels and autophagy may play important roles in the toxicity caused by the CQMa421 culture filtrate.

### 3.4. Transcriptomic Profiling of C. elegans Exposed to the CQMa421 Culture Filtrate

**Figure 5:**
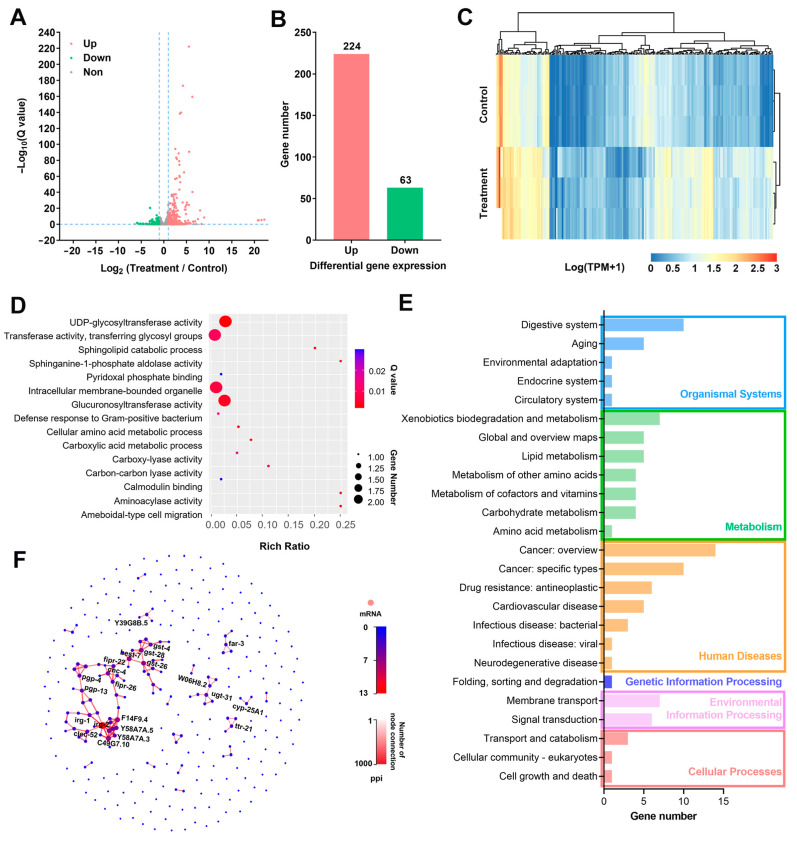
Transcriptomic analysis of DEGs|(log_2_(Treatment/Control)| ≥ 1, Q value ≤ 0.05). (**A**) Differential expression ratios and Q values of DEGs. (**B**) Distribution of upregulated and downregulated genes. (**C**) Heatmap illustrating the normalized expression levels (log_10_(TPM + 1)) of the DEGs. (**D**) Enrichment analyses of GO terms. (**E**) Classification of KEGG pathways. (**F**) PPI network of DEGs, with a minimum score threshold of 500.

To elucidate transcriptomic mechanisms underlying CQMa421 filtrate toxicity, RNA-seq was performed to profile the differential gene expression in *C. elegans*. A transcriptomic analysis identified a total of 18,708 genes, with 224 significantly upregulated and 63 downregulated (Figure 5A–C). To validate transcriptome reliability, 20 differentially expressed genes (DEGs) associated with phenotypic and metabolic alterations were selected for qRT-PCR analysis (Figure S1). The qRT-PCR results correlated with the transcriptome data trends. GO enrichment categorized the DEGs into functional terms including UDP-glycosyltransferase activity, transferase activity, intracellular membrane-bounded organelles and glucuronosyltransferase activity (Figure 5D). The KEGG pathway analysis linked the DEGs to digestion, xenobiotic and lipid metabolism, cancer, drug resistance, membrane transport and signal transduction (Figure 5E). PPI networks constructed using STRING identified key regulatory genes and functional modules in *C. elegans* during filtrate treatment (Figure 5F). Collectively, these GO, KEGG and PPI analyses suggested the potential roles of 53 DEGs in detoxification pathways and immune defense (Table S3).

### 3.5. Transcriptional Basis of CQMa421-Induced Phenotypic and Metabolic Toxicity in C. elegans

**Figure 6:**
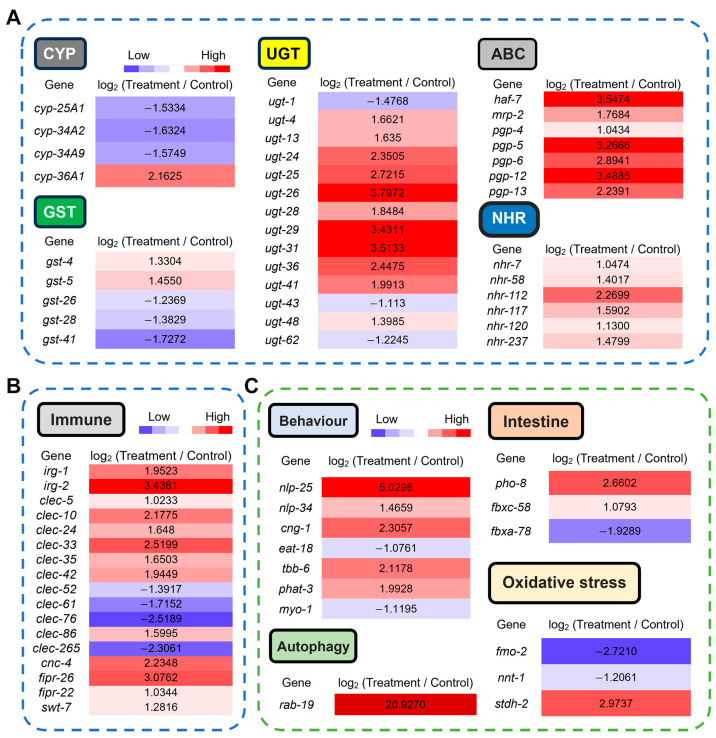
Gene expression profiles of *C. elegans* in response to CQMa421 filtrate treatment. (**A**) Expression patterns of detoxification genes. (**B**) Expression profile of immune-related genes. (**C**) Expression levels of genes associated with locomotor behavior, intestinal function, oxidative stress and autophagy.

The exposure of *C. elegans* to the CQMa421 filtrate significantly impaired survival, locomotor behavior and reproduction, inducing intestinal damage, elevated ROS and autophagy levels. To explore potential pathways underlying these effects, transcriptomic data were analyzed to associate DEGs with phenotypic and metabolic alterations (Figure 6). Mortality in *C. elegans* is theoretically associated with detoxification-related genes and the immune defense system under stress [32,33]. Filtrate exposure modulated detoxification-related genes (Figure 6A), upregulating nuclear hormone receptors (*NHRs*) and ATP-binding cassette transporters (*ABCs*), whereas cytochrome P450 enzymes (*CYPs*), UDP-glucuronosyltransferases (*UGTs*) and glutathione S-transferases (*GSTs*) exhibited variable regulation. Immune-defense-related genes (Figure 6B), including those encoding C-type lectins (e.g., *clec-5* and *clec-10*), an antimicrobial peptide (*cnc-4*), fungus-induced proteins (*fipr-22* and *fipr-26*), a sugar transmembrane transporter (*swt-7*) and infection response genes (*irg-1* and *irg-2*), were predominantly upregulated, with the exception of four C-type lectins (*clec-52*, *clec-61*, *clec-76* and *clec-265*), which were suppressed.

Filtrate exposure suppressed locomotor behavior and reproduction, processes hypothesized to depend on neuromuscular and nervous system activity [34]. Genes putatively critical for neuromuscular function (*eat-18* and *myo-1*) were downregulated, whereas those involved in pharyngeal development (*phat-3*), cytoskeletal structure (*tbb-6*) and neuropeptide signaling (*nlp-25* and *nlp-34*) were upregulated. In addition, intestinal function genes (*pho-8* and *fbxc-58*) were upregulated, while *fbxa-78* was suppressed (Figure 6C).

ROS production has been proposed to play an immune role in *C. elegans*, which may stimulate cellular protective mechanisms and potentially enhance immune defense [35]. Oxidative stress could be caused by imbalances between detoxification pathways and ROS accumulation [36]. Although no phenotypic differences in ROS levels were observed after 6 h of filtrate treatment, a transcriptomic analysis revealed a significant regulation of genes encoding oxidoreductase system components, including monooxygenases (*fmo-2*), transhydrogenases (*nnt-1*), dehydrogenases (*stdh-2*) and detoxification enzymes (*cyp-36A1*, *gst4*, *gst-5* and 11 *UGTs*), hypothesized to be critical for managing oxidative stress. In addition, autophagy is thought to be induced by external stresses and may play a vital role in eliminating toxins and enhancing immune defense [35,37]. Filtrate treatment significantly upregulated *rab-19*, which encodes a GTPase, resulting in a >20-fold increase in its expression (Figure 6C). These findings provide correlative insights into molecular changes associated with nematode mortality and physiological impairment.

## 4. Discussion

Microbial biocontrol agents, including entomopathogenic fungi, hold significant potential for the sustainable management of plant-parasitic nematodes. However, traditional screening methods for nematocidal strains remain labor-intensive, requiring prolonged fermentation, crude extraction and plant-based assays constrained by nematode seasonality and host plant growth cycles [16,38,39]. To address these limitations, we developed a high-throughput screening platform inspired by Lai et al. [18], utilizing *C. elegans* as a model organism to rapidly identify entomopathogenic fungi with nematocidal properties. This platform capitalizes on *C. elegans*’ accessible genomic data, short life cycle and experimental tractability for mechanistic studies of plant-parasitic nematode biology. This approach validated *C. elegans* as an effective proxy for screening, identifying the CQMa421 filtrate as the most potent agent against both *C. elegans* and *M. incognita*. Its effectiveness in controlling plant-parasitic nematodes was further confirmed through pot experiments, which demonstrated a significant alleviation of *M. incognita*-induced damage in *S. lycopersicum*. Moreover, CQMa421, previously registered as a broad-spectrum microbial pesticide effective against insect pests across seven orders [40], demonstrated dual utility for the integrated management of nematodes and insects.

However, this screening method has limitations. While most strains lethal to *C. elegans* exhibited nematocidal activity against *M. incognita*, high lethality in the model nematode did not universally translate into efficacy in plant-parasitic species. Thus, secondary screening against target nematodes remains essential to confirm field applicability. This two-tiered strategy, initial high-throughput screening followed by species-specific validation, ensures the actual functional role while optimizing efficiency for agricultural deployment. It should be emphasized that CQMa421 was prioritized in this screening phase based on its statistically superior lethality. While this empirical approach efficiently narrowed candidates for downstream validation, the biological underpinnings of CQMa421’s enhanced efficacy, such as potential strain-specific toxin production or unique virulence factor expression, require further investigation.

The CQMa421 culture filtrate induced multifaceted toxicity in *C. elegans*, impairing survival, locomotion and reproduction, and causing intestinal damage accompanied by elevated oxidative stress and autophagy levels. These findings collectively suggest the reproductive and neurotoxic effects of the CQMa421 filtrate on nematodes. A transcriptomic analysis linked these phenotypic alterations to dysregulated detoxification pathways and immune defense mechanisms in *C. elegans*, consistent with prior studies highlighting the critical role of detoxification and immune genes in nematode responses to biocontrol agents such as nematocidal metabolites or strains [32,33].

Detoxification genes, including *CYPs*, *GSTs*, *UGTs* and *ABC* transporter family members, are essential for toxin elimination and stress resistance in *C. elegans* [41]. CYPs serve as central mediators in xenobiotic metabolism, influencing the development of toxic effects in *C. elegans* [42]. GSTs and UGTs catalyze protective modifications that neutralize both exogenous and endogenous toxins, thereby maintaining cellular homeostasis [43,44]. ABC transporters, including P-glycoproteins (PGPs), multidrug-resistance-associated proteins (MRPs) and half-transporters (HAFs), serve as cellular safeguards by preventing toxin accumulation and mitigating toxin-induced cellular damage [45]. In parallel, immune effectors such as antimicrobial peptides and proteolytic enzymes have been implicated in *C. elegans* immune responses [35]. For example, irg-1 functions as an immune sentinel [46], while clec-52, cnc-4 and fipr-22 operate as natural or inducible immune effectors [47,48,49], dynamically upregulated in response to external stimuli.

In addition to the involvement of the above genes, processes such as ROS production and autophagy also play roles in *C. elegans* immunity [35]. ROS generation in *C. elegans* is primarily triggered by xenobiotic exposure and cytokine signaling, serving dual functions in immune defense and detoxification mechanisms [50]. During filtrate treatment, ROS production was localized to intestinal regions in *C. elegans*, mirroring patterns observed during *Enterococcus faecalis* infection [51]. Transcriptomic data further revealed a significant upregulation of oxidative-stress-related genes (*fmo-2*, *nnt-1* and *stdh-2*), consistent with oxidative damage mechanisms reported for fosthiazate in nematodes [52]. The intestine, as a primary defense organ in *C. elegans*, plays a vital role in defending against pathogens or toxins, making intestinal damage a crucial indicator of the impact of external stimuli on nematodes [53]. In our study, CQMa421 filtrate exposure likely induced toxin accumulation in intestinal tissues, provoking ROS overproduction and subsequent oxidative stress. While moderate ROS levels function as immune mediators, their excessive accumulation overwhelmed cellular tolerance thresholds, leading to macromolecular damage and mortality.

Autophagy, a conserved cytoprotective process in *C. elegans*, is induced to counteract cytoplasmic insults, including oxidative stress, pathogenic invasion and proteotoxic aggregation [37]. Exposure to the CQMa421 culture filtrate triggered a >20-fold upregulation of *rab-19*, a gene encoding a Rab GTPase central to intracellular trafficking. Rab GTPases orchestrate synaptic vesicle dynamics, immune-related gene expression and membrane fusion events [54], suggesting enhanced endocytosis and membrane fusion are adaptive responses to filtrate exposure. Furthermore, autophagy and oxidative responses function in a synergistic manner to counteract the damage induced by toxins and environmental stressors [55]. The nematotoxicity of the CQMa421 filtrate in *C. elegans* thus stems from multifaceted interactions among detoxification genes, immune effectors and oxidative stress regulators. While transcriptomic correlations align with observed phenotypes, functional validation is needed to establish causality. The causal roles of ROS accumulation and autophagy activation in mediating these effects require validation through pharmacological interventions (e.g., ROS inhibitors like S-allylmercapto-N-acetylcysteine or autophagy blockers such as bafilomycin A1).

Future studies should prioritize LC-MS-based metabolomic profiling and purification of filtrate components to identify key nematocidal compounds, followed by molecular validation (e.g., targeted gene silencing or enzymatic activity assays) to confirm their mode of action. A comparative analysis of CQMa421’s metabolic output against less effective strains could clarify whether its enhanced activity stems from unique secondary metabolites or the differential regulation of conserved virulence pathways. Ultimately, field trials evaluating their efficacy against *M. incognita* in crops will bridge laboratory findings to agricultural applications. The structural elucidation of these metabolites could facilitate the rational design or chemical modification of derivatives with enhanced nematocidal activity. This integrated approach, spanning from compound discovery to field validation, advances *C. elegans* as a screening tool and highlights the potential of entomopathogenic fungi in sustainable nematode management.

## 5. Conclusions

In this study, we established an effective screening platform for nematocidal strains targeting *M. incognita*. Among the 32 entomopathogenic fungal strains, CQMa421 emerged as the most virulent strain, demonstrating both reproductive and neurotoxic effects on nematodes. Transcriptomic profiling linked phenotypic and metabolic effects to the regulation of detoxification pathways, immune responses and oxidative stress mechanisms. These findings highlight the potential of CQMa421 as a dual-action biocontrol agent and validate *C. elegans* as a scalable screening tool for agricultural innovation. Future work should prioritize compound purification, molecular validation and field trials to apply these findings toward sustainable pest management strategies.

## Figures and Tables

**Table 1:** Nematocidal efficacy of entomopathogenic fungi against *C. elegans* and *M. incognita*.

No.	Strains	*C. elegans*				*M. incognita*			
		Mean (%)	SD (%)	Median (%)	Letter Group	Mean (%)	SD (%)	Median (%)	Letter Group
1	SDAY	0.00	0.00	0.00	e	0.00	0.00	0.00	e
2	GB31.000127	3.70	5.43	6.06	e	0.00	0.00	0.00	e
3	GB31.000128	48.06	12.87	53.33	b	2.59	4.13	0.00	e
4	GB3100289	82.22	9.87	85.71	a	58.62	8.23	59.14	a
5	GB3100290	61.02	9.45	63.64	c	14.30	4.01	13.33	d
6	GB3100293	0.00	0.00	0.00	e	2.93	4.02	0.00	e
7	GB3100311	1.11	2.72	0.00	e	23.23	6.82	21.62	c
8	GB3100312	2.41	3.65	0.00	e	0.00	0.00	0.00	e
9	GB3100313	63.54	8.91	63.64	c	53.30	7.94	51.30	a
10	GB3100314	53.95	4.38	53.49	c	26.61	6.45	25.00	c
11	GB3100315	58.60	7.82	55.10	c	44.86	9.73	46.91	b
12	GB3100317	2.74	3.01	3.33	e	0.00	0.00	0.00	e
13	GB3100318	68.75	15.29	68.75	b	23.24	7.12	23.50	c
14	GB3100319	47.52	8.26	44.44	d	58.02	10.89	61.73	a
15	GB3100320	1.85	2.96	0.00	e	0.83	2.04	0.00	e
16	GB3100321	0.79	1.93	0.00	e	32.86	8.32	33.33	c
17	GB3100322	78.33	11.34	83.67	a	0.00	0.00	0.00	e
18	GB3200001	1.41	2.54	0.00	e	0.94	2.31	0.00	e
19	GB3200002	0.67	1.63	0.00	e	1.47	3.60	0.00	e
20	GB3200003	2.36	3.87	0.00	e	4.53	2.76	5.56	e
21	GB3200004	0.00	0.00	0.00	e	0.76	1.85	0.00	e
22	GB3200005	0.93	2.27	0.00	e	0.00	0.00	0.00	e
23	GB3200006	3.28	3.28	2.70	e	0.00	0.00	0.00	e
24	GB3200007	0.00	0.00	0.00	e	0.00	0.00	0.00	e
25	GB3200008	3.87	3.02	3.70	e	0.00	0.00	0.00	e
26	GB3200009	0.00	0.00	0.00	e	4.95	4.12	5.77	e
27	GB3200010	2.09	2.73	0.00	e	0.00	0.00	0.00	e
28	GB3200011	4.76	3.89	4.76	e	17.76	5.33	17.14	c
29	GB3200023	0.00	0.00	0.00	e	0.00	0.00	0.00	e
30	GB3200028	10.02	3.67	9.09	d	3.28	4.03	3.03	e
31	GB3200029	0.00	0.00	0.00	e	0.00	0.00	0.00	e
32	GB3200030	0.52	1.28	0.00	e	0.00	0.00	0.00	e
33	GB3200031	0.00	0.00	0.00	e	0.93	1.63	0.00	e

Non-parametric Kruskal–Wallis test (*p* < 0.001) with Dunn’s post-hoc adjustment for multiple comparisons with five defined efficacy tiers (groups a–e). Shared letters indicate non-significant intergroup differences (*p* > 0.05).

## Data Availability

The RNA-seq data have been uploaded to the NCBI BioProject database with the accession number PRJNA1160025.

## Supplementary Materials

The following supporting information can be downloaded at: https://www.mdpi.com/article/10.3390/jof11050381/s1, Figure S1: qRT–PCR validation of DEGs in the transcriptomic data; Table S1: The primer design of genes in *C. elegans*; Table S2: Statistical Analysis of Experimental Group Comparisons; Table S3: DEGs in *C. elegans* with CQMa421 culture filtrate treatment.
