# Supplementary material for: Biocontrol Potential of Entomopathogenic Fungi Against Plant-Parasitic Nematodes: A Caenorhabditis elegans-Based Screening and Mechanistic Study
Source: J Fungi (Basel). 2025 May 16;11(5):381. doi: 10.3390/jof11050381 (PMC12112724; doi:10.3390/jof11050381)
Supplement: Supplementary file 1 [file jof-11-00381-s001.zip › jof-3617112-supplementary.pdf]

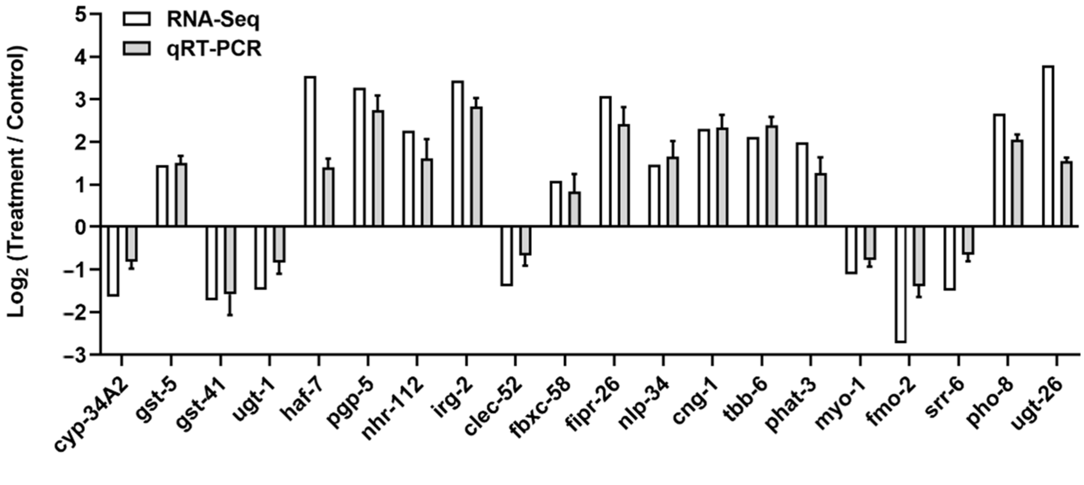

Figure S1. qRT–PCR validation of DEGs in the transcriptomic data. The error bars indicate the SDs.

Table S1. The primer design of genes in *C. elegans*

Gene		Primer
cyp-34A2	Forward	GAGATACGCGCCAGGAATCA
	Reverse	ATGCTCCTGCCAGAATTCCC
gst-5	Forward	TTTGATTGCCAAGGGAGGTG
	Reverse	GAATGGTGTGACTGGACGGG
gst-41	Forward	AGTTCGGGCAAGTTCCATGT
	Reverse	CCCAGGTGTCGCATAATTGC
ugt-1	Forward	ACGCAATTGGGGAACCATCA
	Reverse	AACAGTTTGCTCCGTTCCGA
haf-7	Forward	AAGTGCGGGGAAAAAGGAGT
	Reverse	CAGAACACGTGGATCACGGA
pgp-5	Forward	TCTTCACAGTTTCTGCTTCAATTCT
	Reverse	GCAACAAGGGAAAACTGTCCA
nhr-112	Forward	TAGCAAGGCCTTCCCAACTG
	Reverse	CGGTACTCGAGGATTGGCTC
irg-2	Forward	ACTCAATTCGTGGCCAACCT
	Reverse	TGCCCAGTAACTTGTGTGGG
clec-52	Forward	TGGAGCCCTCTATCAGCAGT
	Reverse	ACTCGGCGGTTTGGAAATCT
fbxc-58	Forward	TCGTGGCCTACTTTGACTGT
	Reverse	TTGACCGAGTCCACCGAATC
fipr-26	Forward	TTTGATGGCCGTTGCTGGTA
	Reverse	CACCTCCACCTAACAGTCCG
nlp-34	Forward	TTCTCCTCATCGCTTGCCTG
	Reverse	ATCCCCATGGGCGGTAGTAT
cng-1	Forward	GCGGTTCGATCCAGATGACA
	Reverse	ATTTTCCGTTGCGAGACCCT
tbb-6	Forward	TCCACTGCAATCCAGGAACC
	Reverse	TTCTCGGCGTCAGTGAACTC
phat-3	Forward	GCTCTGCTAGAGACAGAGATCAT
	Reverse	TTTTCTGTTTCGGCAGTGCTC
myo-1	Forward	GAACACGAGAAGGACCCAGG
	Reverse	TCTTCGAATCGTATGGGCGG
fmo-2	Forward	ACTTGGAACGGTGCCAGAAA
	Reverse	ATTGGTAGGCTCCGGTTTGG
srr-6	Forward	GTTCACTTCCCCCTGTCGTT
	Reverse	CTAGCTGGTCGCAGTGTGAA
pho-8	Forward	GAATTCCGCTTTCCCGATGC
	Reverse	GAGAGGAGTCCCGCATAACG
ugt-26	Forward	TTTGATATTTGCGCACATTGTGGA
	Reverse	AAAACAGTTAATGGGGGAAGTTGT
snb-1	Forward	ATGTGCGCTATCGTCGTCAT
	Reverse	CGGGACAAAGGTCGTGTACT

Table S2. Statistical Analysis of Experimental Group Comparisons

Metric	Normality	Homoscedasticity	Statistical_Test	Significance	Difference
Gall-index	CF: 0.0058 / CK: 0.05	0.48	Mann-Whitney U	<0.0001***	CF<CK
Gall-number	CF: 0.29 / CK: 0.59	0.3	Student’s t-test	0.0004***	CF<CK
Root_Dry_weight	CF: 0.6 / CK: 0.013	0.96	Mann-Whitney U	0.048*	CF>CK
Root_Fresh_weight	CF: 0.75 / CK: 0.62	0.74	Student’s t-test	0.001***	CF>CK
Root_length	CF: 0.83 / CK: 0.76	0.026	Welch’s t-test	0.1728	ns
Stem_Diameter	CF: 0.012 / CK: 0.17	0.18	Mann-Whitney U	0.0279*	CF>CK
Stem_Dry_weight	CF: 1 / CK: 0.45	0.098	Student’s t-test	<0.0001***	CF>CK
Stem_Fresh_weight	CF: 0.52 / CK: 0.27	0.55	Student’s t-test	<0.0001***	CF>CK
Stem_Height	CF: 0.85 / CK: 0.94	0.4	Student’s t-test	<0.0001***	CF>CK
Gall-index	H_2O: 0.031 / CK: 0.05	0.48	Mann-Whitney U	0.808	ns
Gall-number	H_2O: 0.57 / CK: 0.59	0.3	Student’s t-test	0.4659	ns
Root_Dry_weight	H_2O: 0.28 / CK: 0.013	0.96	Mann-Whitney U	0.0573.	ns
Root_Fresh_weight	H_2O: 0.54 / CK: 0.62	0.74	Student’s t-test	0.0308*	H_2O>CK
Root_length	H_2O: 0.75 / CK: 0.76	0.026	Welch’s t-test	0.2525	ns
Stem_Diameter	H_2O: 0.19 / CK: 0.17	0.18	Student’s t-test	0.2732	ns
Stem_Dry_weight	H_2O: 0.8 / CK: 0.45	0.098	Student’s t-test	0.0397*	H_2O>CK
Stem_Fresh_weight	H_2O: 0.61 / CK: 0.27	0.55	Student’s t-test	0.1441	ns
Stem_Height	H_2O: 0.72 / CK: 0.94	0.4	Student’s t-test	0.4913	ns
Mortality_L1	CF: 0.8978 / CK: 0.1438	0.0793	Student’s t-test	<0.0001***	CF>CK
Mortality_L4	CF: 0.8464 / CK: 0.0004	0.0721	Mann-Whitney U	0.00171**	CF>CK
Mortality_YA	CF: 0.9795 / CK: 0	0.0275	Mann-Whitney U	0.00141**	CF>CK
Mortality_D1	CF: 0.9747 / CK: 0.0142	0.1602	Mann-Whitney U	0.00193**	CF>CK
egg_laying	CF: 0.5486 / CK: 0.3911	0.3556	Student’s t-test	0.0013**	CF<CK
hatching	CF: 0.5155 / CK: 0.9529	0.3165	Student’s t-test	0.008**	CF<CK
Survival_Curve_6h	-	-	Mann-Whitney U	-	ns
Survival_Curve_12h	CF: 0.47 / CK: 0.038	0.025	Mann-Whitney U	0.011*	CF<CK
Survival_Curve_18h	CF: 0.8 / CK: 0.039	0.02	Mann-Whitney U	0.011*	CF<CK
Survival_Curve_24h	CF: 1 / CK: 0.36	0.096	Student's t-test	<0.0001***	CF<CK
Survival_Curve_30h	CF: 0.85 / CK: 0.69	0.14	Student's t-test	<0.0001***	CF<CK
Survival_Curve_36h	CF: 0.031 / CK: 0.14	0.89	Mann-Whitney U	0.011*	CF<CK
Head_2h	CF: 0.0197 / CK: 0.6757	0.5488	Mann-Whitney U	0.449	ns
Head_4h	CF: 0.1486 / CK: 0.8676	0.4646	Student’s t-test	0.1752	ns
Head_6h	CF: 0.4507 / CK: 0.153	0.5316	Student’s t-test	0.0018**	CF<CK
Head_12h	CF: 0.787 / CK: 0.5667	0.6592	Student’s t-test	<0.0001***	CF<CK
Pumping_2h	CF: 0.0913 / CK: 0.7899	0.0687	Student’s t-test	<0.0001***	CF<CK
Pumping_4h	CF: 0.4575 / CK: 0.1028	0.7742	Student’s t-test	<0.0001***	CF<CK
Pumping_6h	CF: 0.0362 / CK: 0.7228	1	Mann-Whitney U	0.000179***	CF<CK
Pumping_12h	CF: 0.4333 / CK: 0.9369	0.436	Student’s t-test	<0.0001***	CF<CK
Recover_Head_2h	CF: 0.918 / CK: 0.1525	0.5028	Student’s t-test	0.5843	ns
Recover_Head_4_h	CF: 0.2798 / CK: 0.4646	0.0209	Welch’s t-test	0.9045	ns
Recover_Head_6_h	CF: 0.1992 / CK: 0.0589	0.172	Student’s t-test	0.1377	ns

Metric	Normality	Homoscedasticity	Statistical_Test	Significance	Difference
Recover_Head_12h	CF: 0.7969 / CK: 0.2999	0.3804	Student’s t-test	0.007**	CF<CK
Recover_Pumping_2h	CF: 0.0003 / CK: 0.3204	0.1755	Mann-Whitney U	0.384	ns
Recover_Pumping_4h	CF: 0.0178 / CK: 0.9137	0.0936	Mann-Whitney U	0.256	ns
Recover_Pumping_6h	CF: 0.0153 / CK: 0.9619	0	Mann-Whitney U	0.0587.	ns
Recover_Pumping_12h	CF: 0.1028 / CK: 0.1882	0.0266	Welch’s t-test	0.0002***	CF<CK
ROS_6h	CF: 0.898 / CK: 0.0632	0.1271	Student’s t-test	0.6169	ns
ROS_12h	CF: 0.9752 / CK: 0.1631	0.178	Student’s t-test	0.0002***	CF>CK
ROS_18h	CF: 0.1778 / CK: 0.6642	0.0111	Welch’s t-test	0.0048**	CF>CK
ROS_24h	CF: 0.7846 / CK: 0.791	0.0074	Welch’s t-test	0.0057**	CF>CK
Nile_6h	CF: 0.0373 / CK: 0.2437	0.0451	Mann-Whitney U	0.27	ns
Nile_12h	CF: 0.8906 / CK: 0.1978	0.453	Student’s t-test	0.1214	ns
Nile_18h	CF: 0.8653 / CK: 0.1238	0.2295	Student’s t-test	0.0007***	CF>CK
Nile_24h	CF: 0.7235 / CK: 0.0226	0.3216	Mann-Whitney U	0.00388**	CF>CK
AL	CF: 0.0832 / CK: 0.0012	0	Mann-Whitney U	<0.0001***	CF>CK
AP	CF: 0.0425 / CK: 0	0.1467	Mann-Whitney U	<0.0001***	CF>CK

**p* <0.05, ***p* <0.01, ****p* <0.001, ns indicates nonsignificant.

Table S3. DEGs in *C. elegans* with CQMa421 culture filtrate treatment

Gene	$\log_2$ (Treatment / Control)	Q value (Treatment / Control)	Control	Treatment	GeneBank Description
abf-5	1.2372	0.0004	7.0133	16.3367	AntiBacterial Factor related
abhd-5.2	1.1461	0.0000	15.1800	32.4467	Abhydrolase domain-containing protein abhd-5.2
amt-1	3.5487	0.0001	0.1767	2.0767	Putative ammonium transporter 1
amt-4	−1.3166	0.0000	15.7800	6.2867	Ammonium transporter
arf-1.1	1.3891	0.0002	6.4867	16.7867	ADP-ribosylation factor 1-like 1
B0205.13	2.3871	0.0000	4.1000	21.0400	Uncharacterized protein
B0507.2	22.1945	0.0000	0.0000	1.4367	BROX homolog
B0554.5	1.2798	0.0001	2.4667	5.8833	Uncharacterized protein
best-1	1.3745	0.0000	4.0367	10.3467	Bestrophin homolog
best-7	1.5012	0.0188	2.5800	7.0967	Bestrophin homolog
C01B4.8	1.4867	0.0116	1.3700	3.8633	MFS domain-containing protein
C01G10.17	5.7097	0.0157	0.0767	4.2733	Uncharacterized protein
C01G10.4	6.5823	0.0004	0.0867	9.2200	Uncharacterized protein
C01G10.5	6.4342	0.0006	0.0800	7.5600	Uncharacterized protein
C03G6.17	−1.3611	0.0004	12.3200	4.7900	DUF1248 domain-containing protein
C04A11.5	2.6307	0.0000	2.1267	13.0367	Uncharacterized protein
C05D11.5	1.2388	0.0002	5.1467	11.8833	Putative hydroxypyruvate isomerase
C06B3.6	1.4159	0.0000	54.3567	144.0333	Uncharacterized protein
C06B3.7	5.5899	0.0000	0.8367	39.9000	Uncharacterized protein
C10C5.3	1.6750	0.0000	6.4100	19.9767	N-acyl-L-amino-acid amidohydrolase
C16E9.1	−1.5915	0.0299	2.8300	0.9533	Uncharacterized protein
C17E4.10	3.3005	0.0374	0.2567	5.8167	Uncharacterized protein
C18D11.6	1.8829	0.0226	1.8900	6.8733	Uncharacterized protein
C18H7.1	−1.4577	0.0126	9.0967	3.5000	VWFA domain-containing protein
C25H3.10	−1.1677	0.0000	18.1867	7.7933	F-box domain-containing protein
C27B7.9	1.3080	0.0000	90.4933	220.7333	Uncharacterized protein
C28C12.4	2.7366	0.0000	1.3800	8.9400	DUF148 domain-containing protein
C29F3.7	−1.1457	0.0045	46.6267	21.0300	CUB_2 domain-containing protein
C37A5.3	1.2804	0.0008	1.7067	4.0400	Uncharacterized protein
C45B11.2	2.0266	0.0000	1.2000	4.8233	Uncharacterized protein
C49F8.1	1.5859	0.0000	4.1133	12.2500	Uncharacterized protein
C49G7.10	2.1400	0.0000	26.4500	113.6367	Uncharacterized protein
C49G7.12	1.0293	0.0012	10.7333	21.5267	Uncharacterized protein
C50D2.6	1.0120	0.0008	9.1967	19.6033	CPG4 domain-containing protein
C50F7.5	1.4377	0.0000	146.5167	392.4167	Uncharacterized protein
C52B11.5	1.0145	0.0011	8.2133	16.3067	Uncharacterized protein
catp-3	2.4811	0.0000	20.8833	113.8000	Cation_ATPase_N domain-containing protein

Gene	$\log_2$ (Treatment / Control)	Q value (Treatment / Control)	Control	Treatment	GeneBank Description
cdr-2	−1.4998	0.0000	17.8167	6.2133	CaDmium Responsive
cdr-4	2.1374	0.0000	10.1333	43.3900	CaDmium Responsive
cest-12	1.1780	0.0009	2.2600	5.0067	Carboxyl ESTerase domain containing
cest-34	3.4312	0.0000	1.3200	14.1500	Carboxylic ester hydrolase
cest-9.1	−1.1364	0.0076	3.0500	1.3733	COesterase domain-containing protein
clec-10	2.1775	0.0000	7.5700	33.4400	C-type LECtin
clec-24	1.6480	0.0024	1.6900	5.1400	C-type LECtin
clec-265	−2.3061	0.0004	18.3667	3.7233	C-type LECtin
clec-33	2.5199	0.0000	0.4500	2.5533	C-type LECtin
clec-35	1.6503	0.0429	1.1500	3.5567	C-type LECtin
clec-42	1.9449	0.0009	0.6467	2.3967	C-type LECtin
clec-5	1.0233	0.0000	34.9033	70.0467	C-type LECtin
clec-52	−1.3917	0.0000	36.2700	13.8033	C-type LECtin
clec-61	−1.7152	0.0413	3.7833	1.1500	C-type LECtin
clec-76	−2.5189	0.0000	2.5900	0.4567	C-type LECtin
clec-86	1.5995	0.0001	8.4067	25.4933	C-type LECtin
clp-9	1.0442	0.0001	2.6600	5.5267	Calpain catalytic domain-containing protein
cnc-4	2.2348	0.0000	43.2100	200.0333	CaeNaCin
cng-1	2.3057	0.0000	2.9200	14.1667	Cyclic nucleotide-binding domain-containing protein
col-95	−1.0690	0.0001	206.7733	98.1300	Col_cuticle_N domain-containing protein
cul-6	2.2485	0.0000	3.0733	14.3700	Cullin-6
cyp-13A10	2.2825	0.0000	3.1400	14.9333	Putative cytochrome P450 CYP13A10
cyp-14A5	2.2617	0.0000	33.2667	157.9067	CYtochrome P450 family
cyp-25A1	−1.5334	0.0002	4.4267	1.5233	CYtochrome P450 family
cyp-25A2	−1.1099	0.0274	10.0933	4.6733	CYtochrome P450 family
cyp-34A2	−1.6324	0.0117	4.4000	1.4000	CYtochrome P450 family
cyp-34A9	−1.5749	0.0000	59.5933	18.8133	CYtochrome P450 family
cyp-36A1	2.1625	0.0000	2.8667	12.6300	Putative cytochrome P450 CYP36A1
dct-7	1.7709	0.0303	8.6800	29.6967	DAF-16/FOXO Controlled, germline Tumor affecting
dhs-15	−3.1046	0.0006	2.8900	0.3300	DeHydrogenases, Short chain
dhs-2	−1.3609	0.0003	18.8100	7.1267	DeHydrogenases, Short chain
dhs-20	−1.6223	0.0153	6.5500	2.1300	DeHydrogenases, Short chain
dod-23	2.0793	0.0000	334.5233	1392.7633	Downstream Of DAF-16
dod-3	−2.0194	0.0027	29.1600	7.2400	Downstream Of DAF-16

Gene	log_2 (Treatment / Control)	Q value (Treatment / Control)	Control	Treatment	GeneBank Description
dos-3	3.9077	0.0080	0.0433	0.5267	Delta and OSM-11-like
drd-1	1.2909	0.0004	4.2667	10.3733	Fatty acid hydroxylase domain-containing protein
E02H4.7	−1.5374	0.0000	14.5833	4.9667	Uncharacterized protein
E03H4.8	1.1669	0.0000	28.6467	63.6633	Coatomer_WDAD domain-containing protein
eat-18	−1.0761	0.0448	3.2300	1.4567	Uncharacterized protein
elo-5	1.1621	0.0000	31.7567	70.0433	Elongation of long chain fatty acids protein
F09C6.1	4.8253	0.0000	0.2567	7.1733	Inducible FAScin Domain containing
F09C6.14	5.3616	0.0000	0.1033	4.0633	Uncharacterized protein
F09C6.3	5.5464	0.0000	0.3433	15.8933	Uncharacterized protein
F10C2.7	3.5228	0.0001	0.2133	2.4067	MFS domain-containing protein
F10D7.3	1.9169	0.0000	5.3267	19.6967	putative monothiol glutaredoxin F10D7.3
F11E6.11	1.4588	0.0001	9.0633	22.9767	Uncharacterized protein
F13A7.11	1.1062	0.0001	14.9700	31.5667	Uncharacterized protein
F13H8.11	−1.0861	0.0309	3.3300	1.5600	Lipase_GDSL domain-containing protein
F14F9.3	6.3268	0.0000	0.1767	13.2867	Uncharacterized protein
F14F9.4	4.2157	0.0000	2.9533	53.9367	Uncharacterized protein
F14H12.3	2.2877	0.0000	3.1167	14.9267	Uncharacterized protein
F17C11.4	1.0279	0.0000	49.5233	99.7433	Uncharacterized protein
F19C7.2	1.9397	0.0000	13.8833	52.2800	Uncharacterized protein
F22F7.8	1.4230	0.0009	11.8200	30.8100	Uncharacterized protein
F22H10.2	2.0184	0.0000	13.4333	54.0500	Uncharacterized protein
F25D1.5	2.5726	0.0002	2.6400	15.1033	Uncharacterized protein
F28F5.4	1.6701	0.0247	2.6633	8.4467	Uncharacterized protein
F35C11.6	1.1789	0.0192	3.5100	7.8533	Uncharacterized protein
F38B7.2	1.5819	0.0000	3.1333	9.1667	CX domain-containing protein
F41C3.2	1.2056	0.0002	4.1767	9.4967	Uncharacterized protein
F43C1.7	4.4737	0.0000	2.1467	47.3200	Coiled-coil domain-containing protein
F48C1.9	1.5915	0.0080	3.2767	9.6300	Uncharacterized protein
F52E4.5	1.0011	0.0000	9.5267	18.7600	Uncharacterized protein
F53A9.9	1.0832	0.0215	7.4700	15.5100	Uncharacterized protein
F53F1.6	1.6222	0.0478	2.7467	8.1600	Uncharacterized protein
F54B8.4	2.2850	0.0000	25.9567	127.4467	Uncharacterized protein
F55G11.8	−1.2728	0.0314	9.1800	3.8067	CUB_2 domain-containing protein
F56D2.5	2.4794	0.0000	5.2367	28.7667	RBR-type E3 ubiquitin transferase

Gene	$\log_2$ (Treatment / Control)	Q value (Treatment / Control)	Control	Treatment	GeneBank Description
F56D5.3	2.6053	0.0000	0.4933	2.9667	Oxidored_FMN domain-containing protein
F58G6.7	−1.5122	0.0317	4.8067	1.6900	Copper transporter
F59B1.10	1.5200	0.0262	0.7867	2.1867	CHK domain-containing protein
far-3	2.2014	0.0000	20.3300	91.2767	Fatty Acid/Retinol binding protein
fbxa-111	1.4737	0.0080	0.6267	1.7167	F-box domain-containing protein
fbxa-128	1.7586	0.0000	3.1933	10.6600	FTH domain-containing protein
fbxa-143	1.3495	0.0273	1.5733	3.9267	F-box domain-containing protein
fbxa-37	1.8453	0.0000	5.0367	17.8833	F-box domain-containing protein
fbxa-44	4.8955	0.0000	0.2067	6.0967	F-box domain-containing protein
fbxa-50	2.7717	0.0000	0.8900	5.9367	F-box A protein
fbxa-71	1.6768	0.0078	0.8233	2.5467	FTH domain-containing protein
fbxa-78	−1.9289	0.0276	2.0267	0.5267	F-box domain-containing protein
fbxc-1	20.7753	0.0000	0.0000	0.8067	F-box C protein
fbxc-58	1.0793	0.0000	14.7500	30.8100	F-box C protein
fip-6	1.9197	0.0051	4.5967	16.7300	Fungus-Induced Protein
fipr-22	1.0344	0.0031	6.3700	12.9100	Fungus-Induced Protein Related
fipr-26	3.0762	0.0000	2.5833	21.6300	Fungus-Induced Protein Related
fmo-2	−2.7210	0.0000	406.1533	61.5333	Dimethylaniline monooxygenase
fpn-1.2	2.9115	0.0000	1.1567	8.5233	Solute carrier family 40 protein
glb-33	1.5592	0.0000	3.3100	9.4833	GLoBin related
gst-12	2.7092	0.0000	3.7900	24.1067	Glutathione S-Transferase
gst-16	1.1282	0.0009	1.9100	4.1133	Glutathione S-Transferase
gst-26	−1.2369	0.0294	7.5900	3.2400	Glutathione S-Transferase
gst-28	−1.3829	0.0001	10.8000	4.1567	Glutathione S-Transferase
gst-31	2.6597	0.0000	1.2867	8.0600	Glutathione S-Transferase
gst-4	1.3304	0.0000	7.4767	18.5433	Glutathione S-transferase
gst-41	−1.7272	0.0070	5.4167	1.6267	Glutathione S-Transferase
gst-5	1.4550	0.0000	41.6867	103.0967	Glutathione S-transferase
H02F09.3	2.9683	0.0001	0.2300	1.8300	Uncharacterized protein
H06H21.8	1.3427	0.0000	19.5500	48.6367	CHK domain-containing protein
haf-7	3.5474	0.0000	7.0133	81.2133	HAlF transporter (PGP related)
hsp-17	1.3688	0.0000	12.8900	32.8267	SHSP domain-containing protein
igeg-2	1.3193	0.0033	1.6567	4.0967	IG (immunoglobulin), EGF and transmmembrane domain
ilys-3	1.2485	0.0001	27.6400	65.4133	Invertebrate-type lysozyme 3
ins-7	1.4009	0.0001	21.0633	54.5733	Insulin-like peptide
ins-8	8.9036	0.0000	0.0000	16.3600	Insulin related
irg-1	1.9523	0.0000	7.3300	28.1033	NADAR domain-containing protein
irg-2	3.4361	0.0000	7.3733	78.3933	Infection Response Gene

Gene	$\log_2$ (Treatment / Control)	Q value (Treatment / Control)	Control	Treatment	GeneBank Description
K08D10.14	1.2772	0.0001	10.5467	25.0000	Uncharacterized protein
K08F9.1	−1.4769	0.0000	8.8933	3.1700	MFS domain-containing protein
K08F9.3	−1.2798	0.0058	5.7933	2.4267	Uncharacterized protein
K09D9.1	2.1178	0.0000	5.6767	24.4400	Uncharacterized protein
K09F6.10	1.9944	0.0000	1.4867	5.8033	Uncharacterized protein
K09F6.13	1.1819	0.0052	2.4867	5.6067	Uncharacterized protein
K09F6.6	2.8908	0.0231	0.1400	0.9500	Uncharacterized protein
K10H10.9	7.5364	0.0000	0.0333	6.7633	Uncharacterized protein
lec-7	1.5165	0.0050	1.8733	5.2600	Putative galaptin lec-7
M03A1.8	2.6378	0.0000	5.3833	33.0400	Cytochrome b561 domain-containing protein
M162.5	1.7752	0.0034	3.6933	12.2600	MFS domain-containing protein
M176.4	1.2548	0.0000	20.4467	48.1500	Uncharacterized protein
M60.7	1.4453	0.0000	5.7433	15.3867	Uncharacterized protein
mrp-2	1.7684	0.0000	3.5333	11.9000	Uncharacterized protein
msp-59	2.2796	0.0061	2.0000	9.0633	Major sperm protein
mtl-1	−2.2413	0.0038	516.5000	109.6100	Metallothionein-1
mxl-3	1.5806	0.0016	5.8100	17.2500	Protein mxl-3
myo-1	−1.1195	0.0001	11.9967	5.5033	Myosin-1
nhr-112	2.2699	0.0000	3.1100	14.7900	Nuclear Hormone Receptor family
nhr-117	1.5902	0.0000	3.2133	9.4033	Nuclear Hormone Receptor family
nhr-120	1.1300	0.0000	6.5900	14.2200	Nuclear Hormone Receptor family
nhr-237	1.4800	0.0282	1.6933	4.6100	Nuclear Hormone Receptor family
nhr-58	1.4017	0.0000	8.1800	21.3033	Nuclear Hormone Receptor family
nhr-7	1.0474	0.0349	1.3733	2.7867	Nuclear Hormone Receptor family
nlp-25	5.0296	0.0000	0.5067	16.1867	Neuropeptide-Like Protein
nlp-34	1.4659	0.0200	5.5133	15.0900	Neuropeptide-Like Protein
nlp-73	1.2751	0.0000	14.3500	34.1433	Neuropeptide-Like Protein
nnt-1	−1.2061	0.0000	23.2900	10.0267	Proton-translocating NAD(P)(+) transhydrogenase
oac-53	1.7231	0.0033	0.5567	1.8100	NRF domain-containing protein
oac-54	5.1029	0.0000	0.5600	18.3500	Acyl_transf_3 domain-containing protein
oac-7	2.8754	0.0000	1.1467	8.3733	NRF domain-containing protein
otpl-5	4.8071	0.0014	0.0367	0.9333	OToPetrin-Like
otpl-6	4.6233	0.0000	0.1767	4.2333	OToPetrin-Like
pals-24	−1.4209	0.0011	4.5567	1.6867	Protein containing ALS2cr12 (ALS2CR12) signature
pals-32	1.4220	0.0005	4.6867	12.5067	Protein containing ALS2cr12 (ALS2CR12) signature
parg-2	2.8512	0.0000	6.0033	42.6233	Poly(ADP-ribose) glycohydrolase

Gene	log_2 (Treatment / Control)	Q value (Treatment / Control)	Control	Treatment	GeneBank Description
pgp-12	3.4885	0.0000	0.2067	2.2133	P-GlycoProtein related
pgp-13	2.2391	0.0000	0.6567	3.0833	P-GlycoProtein related
pgp-4	1.0434	0.0001	2.0433	4.1100	P-GlycoProtein related
pgp-5	3.2666	0.0000	2.2167	21.3033	P-GlycoProtein related
pgp-6	2.8941	0.0000	9.0233	66.3033	P-GlycoProtein related
pgp-7	2.9625	0.0000	0.2333	1.7333	P-GlycoProtein related
phat-3	1.9928	0.0000	5.0867	19.9233	PHAryngeal gland Toxin-related
pho-8	2.6602	0.0000	0.7367	4.5133	intestinal acid PHOsphatase
pitr-4	2.2568	0.0390	0.8100	3.6600	Phosphate transporter
pqn-31	2.3293	0.0000	5.7100	28.0633	Prion-like-(Q/N-rich)-domain-bearing protein
pqn-36	1.0131	0.0013	2.4567	4.6767	Prion-like-(Q/N-rich)-domain-bearing protein
pqn-54	2.1609	0.0002	0.6733	2.8867	Prion-like-(Q/N-rich)-domain-bearing protein
R02C2.7	−3.0299	0.0001	13.4167	1.5900	DUF4440 domain-containing protein
R05D8.7	1.1268	0.0153	4.1133	8.7833	Uncharacterized protein
R07C12.1	3.6275	0.0000	5.1533	62.2600	Uncharacterized protein
R07C12.3	2.4163	0.0402	0.4300	2.2500	Uncharacterized protein
R08F11.4	3.4066	0.0000	3.8833	40.7067	Methyltranfer_dom domain-containing protein
R09H10.5	−1.0718	0.0060	13.2267	6.2800	Uncharacterized protein
R10E4.7	−5.9088	0.0187	0.3167	0.0000	Uncharacterized protein
rab-19	20.9270	0.0000	0.0000	4.1567	RAB family
rfs-1	21.5502	0.0000	0.0000	1.3033	RAD51-like protein
sir-2.3	1.7923	0.0002	1.4833	5.0800	NAD-dependent protein deacylase
slc-17.5	2.0045	0.0000	1.6633	6.5567	MFS domain-containing protein
slc-36.5	−1.4886	0.0006	9.7133	3.4367	Aa_trans domain-containing protein
spl-2	2.1591	0.0000	3.6133	15.7767	Sphingosine Phosphate Lyase
spp-2	−1.2839	0.0000	83.0933	33.8433	Saposin B-type domain-containing protein
srr-4	1.3296	0.0000	16.4767	40.7900	Serpentine Receptor
srr-6	−1.5000	0.0048	5.0300	1.7633	Serpentine Receptor
stdh-2	2.9737	0.0046	0.2500	1.7433	Putative steroid dehydrogenase
str-144	3.3074	0.0000	0.3333	3.2333	Seven TM Receptor
sulp-8	1.4868	0.0000	12.0667	33.4967	STAS domain-containing protein
swt-2	5.8271	0.0180	0.0000	1.0000	Sugar transporter
swt-7	1.2816	0.0000	46.8267	112.2233	Sugar transporter
T05E12.3	2.5353	0.0000	28.0233	160.8500	BTB domain-containing protein
T05F1.11	−1.2882	0.0493	2.1467	0.8733	Thioredoxin domain-containing protein

Gene	$\log_2$ (Treatment / Control)	Q value (Treatment / Control)	Control	Treatment	GeneBank Description
T05F1.9	2.2188	0.0000	0.9333	4.3033	Uncharacterized protein
T07H8.11	4.6798	0.0001	0.0600	1.4833	Uncharacterized protein
T08B2.12	6.9179	0.0002	0.0000	2.2433	Uncharacterized protein
T10C6.15	1.5003	0.0004	3.0133	5.6433	F-box domain-containing protein
T12D8.5	−1.3854	0.0000	39.3167	14.9700	Uncharacterized protein
T16G1.4	1.3676	0.0317	2.8700	7.2067	CHK domain-containing protein
T16G1.5	4.1533	0.0000	0.4200	7.3533	CHK domain-containing protein
T19C9.8	−2.1187	0.0085	12.3667	2.8567	Uncharacterized protein
T24A6.7	4.9948	0.0000	0.3367	10.8033	NADAR domain-containing protein
T27F6.8	1.4408	0.0000	12.2567	32.8267	F-box domain-containing protein
T28D6.3	−2.5752	0.0024	4.3233	0.7267	Uncharacterized protein
tbb-6	2.1178	0.0000	9.8067	41.9400	Tubulin beta chain
thn-1	2.6256	0.0000	1.8933	11.3400	THaumatiN family
thn-2	1.3788	0.0000	88.0067	224.1767	THaumatiN family
tnc-2	−1.0873	0.0011	29.4967	13.8233	Troponin C
tre-5	1.5184	0.0000	3.0167	8.5867	Trehalase
trx-3	2.0092	0.0000	14.0667	55.4467	Thioredoxin domain-containing protein
tsp-3	1.3660	0.0006	3.7567	9.5100	TetraSPanin family
ttc-36	1.1653	0.0000	19.0400	42.1233	Tetratricopeptide repeat protein
ttr-21	1.9884	0.0000	4.6600	18.1333	TransThyretin-Related
ttr-26	2.7459	0.0000	25.6700	170.0200	TransThyretin-Related
ttr-30	1.1104	0.0000	18.7133	39.6200	TransThyretin-Related
ttr-44	−1.8711	0.0071	28.6567	8.1267	TransThyretin-Related
twk-33	−1.0504	0.0133	3.2433	1.5533	TWiK family of potassium channels
ugt-1	−1.4769	0.0005	7.7900	2.7933	Glucuronosyltransferase
ugt-13	1.6351	0.0000	2.3700	7.2067	Glucuronosyltransferase
ugt-24	2.3505	0.0000	2.6067	13.1200	UDP-glucuronosyltransferase
ugt-25	2.7215	0.0000	3.4033	21.8733	UDP-glucuronosyltransferase
ugt-26	3.7972	0.0000	5.8167	79.7633	UDP-glucuronosyltransferase
ugt-28	1.8484	0.0000	5.4200	19.1567	Glucuronosyltransferase
ugt-29	3.4311	0.0000	9.9800	106.4300	Glucuronosyltransferase
ugt-31	3.5133	0.0000	3.3933	37.8367	Glucuronosyltransferase
ugt-36	2.4475	0.0019	0.7733	4.0433	Glucuronosyltransferase
ugt-4	1.6622	0.0000	6.1033	18.9633	UDP-glucuronosyltransferase
ugt-41	1.9913	0.0062	1.8800	7.1767	Glucuronosyltransferase
ugt-43	−1.1130	0.0347	3.5267	1.5767	Glucuronosyltransferase
ugt-48	1.3985	0.0000	5.4867	14.2967	UDP-glucuronosyltransferase
ugt-62	−1.2246	0.0002	28.9767	12.2800	UDP-glucuronosyltransferase
W02D7.11	−2.8780	0.0000	16.3900	2.2533	Uncharacterized protein
W02D9.10	1.0860	0.0000	26.6667	56.1433	Uncharacterized protein

Gene	log_2 (Treatment / Control)	Q value (Treatment / Control)	Control	Treatment	GeneBank Description
W03F8.6	−1.1103	0.0121	39.5233	18.8533	Uncharacterized protein
W06H8.2	1.5021	0.0004	1.4333	4.0233	Oxidored_FMN domain-containing protein
Y17D7B.2	4.7632	0.0000	0.1600	4.2767	Uncharacterized protein
Y17D7B.3	8.0236	0.0000	0.0800	21.2033	Uncharacterized protein
Y19D10A.5	1.4867	0.0116	1.3700	3.8633	MFS domain-containing protein
Y39A3A.4	2.6005	0.0000	1.3467	8.0300	Tyrosine-protein phosphatase domain-containing protein
Y39B6A.27	1.1576	0.0013	2.9300	6.4367	Uncharacterized protein
Y39G8B.5	3.4230	0.0368	0.0600	0.5833	Protein kinase domain-containing protein
Y42A5A.3	1.2414	0.0128	6.5633	15.2233	Uncharacterized protein
Y42G9A.3	1.2855	0.0000	11.1700	26.7933	Uncharacterized protein
Y45F10D.6	2.3890	0.0000	7.9000	40.4300	Uncharacterized protein
Y45G5AM.3	1.0904	0.0000	18.1667	38.4067	Uncharacterized protein
Y46C8AM.1	−1.6161	0.0094	3.7933	1.2400	C-type lectin domain-containing protein
Y47H10A.3	−1.7132	0.0056	10.2467	3.1200	Decapping nuclease
Y47H9C.1	−1.2591	0.0161	8.0933	3.3767	Uncharacterized protein
Y51F10.7	−1.6650	0.0000	286.8733	90.3233	Uncharacterized protein
Y53F4B.1	1.0961	0.0016	2.0267	4.2800	Protein kinase domain-containing protein
Y57E12B.11	5.2893	0.0222	0.0767	3.4400	Uncharacterized protein
Y57E12B.4	5.8119	0.0391	0.0000	0.6433	Uncharacterized protein
Y58A7A.3	1.7185	0.0000	35.1767	114.2033	Uncharacterized protein
Y58A7A.4	5.5490	0.0000	1.3100	59.5167	Uncharacterized protein
Y58A7A.5	6.3061	0.0000	1.5533	120.4467	Uncharacterized protein
Y60C6A.3	5.0436	0.0354	0.0633	2.3600	Uncharacterized protein
Y6E2A.4	−2.1398	0.0006	5.7900	1.3267	Uncharacterized protein
Y6G8.2	−1.8065	0.0076	3.1467	0.9000	Uncharacterized protein
Y73F4A.2	−3.0237	0.0000	38.7133	4.7567	DOMON domain-containing protein
ZC266.1	1.0878	0.0038	5.9967	12.4833	Uncharacterized protein
ZC395.5	−1.0899	0.0075	52.6767	24.6567	Uncharacterized protein
ZK1290.5	1.2218	0.0472	3.3433	7.6367	putative oxidoreductase
ZK228.3	1.0365	0.0162	5.9267	11.9233	Acetyltransf_18 domain-containing protein
ZK228.4	3.0828	0.0000	2.8367	23.1933	Uncharacterized protein
ZK287.9	1.1652	0.0004	12.1733	26.8300	Uncharacterized protein
ZK970.7	1.9957	0.0009	4.6600	18.3800	DUF148 domain-containing protein
